# Accurate Inference of Subtle Population Structure (and Other Genetic Discontinuities) Using Principal Coordinates

**DOI:** 10.1371/journal.pone.0004269

**Published:** 2009-01-27

**Authors:** Patrick A. Reeves, Christopher M. Richards

**Affiliations:** United States Department of Agriculture, Agricultural Research Service, National Center for Genetic Resources Preservation, Fort Collins, Colorado, United States of America; University of Chicago, United States of America

## Abstract

**Background:**

Accurate inference of genetic discontinuities between populations is an essential component of intraspecific biodiversity and evolution studies, as well as associative genetics. The most widely-used methods to infer population structure are model-based, Bayesian MCMC procedures that minimize Hardy-Weinberg and linkage disequilibrium within subpopulations. These methods are useful, but suffer from large computational requirements and a dependence on modeling assumptions that may not be met in real data sets. Here we describe the development of a new approach, PCO-MC, which couples principal coordinate analysis to a clustering procedure for the inference of population structure from multilocus genotype data.

**Methodology/Principal Findings:**

PCO-MC uses data from all principal coordinate axes simultaneously to calculate a multidimensional “density landscape”, from which the number of subpopulations, and the membership within subpopulations, is determined using a valley-seeking algorithm. Using extensive simulations, we show that this approach outperforms a Bayesian MCMC procedure when many loci (e.g. 100) are sampled, but that the Bayesian procedure is marginally superior with few loci (e.g. 10). When presented with sufficient data, PCO-MC accurately delineated subpopulations with population F_st_ values as low as 0.03 (G'_st_>0.2), whereas the limit of resolution of the Bayesian approach was F_st_ = 0.05 (G'_st_>0.35).

**Conclusions/Significance:**

We draw a distinction between population structure inference for describing biodiversity as opposed to Type I error control in associative genetics. We suggest that discrete assignments, like those produced by PCO-MC, are appropriate for circumscribing units of biodiversity whereas expression of population structure as a continuous variable is more useful for case-control correction in structured association studies.

## Introduction

Genetically structured populations arise when gene flow between groups of individuals is hindered by geographical, behavioral, temporal, or genomic barriers. The identification of natural groups of individuals that have originated due to partial reproductive barriers has become an important component of contemporary evolutionary biology research. The existence of such groups promotes divergence and, ultimately, the origin of evolutionary novelties. The presence of genetic structure in natural populations, and its role as an engine of diversification, is an essential element of modern evolutionary theory.

The failure to recognize population genetic structure has serious ecological implications. When cryptic subpopulations go unnoticed, *in situ* conservation efforts are compromised [1], [2]. Failure to properly infer genetic structure can result in the quiet disappearance of important, but hidden, contributors to ecosystem diversity [3]–[5]. The *ex situ* conservation efforts of zoos, botanical gardens, or germplasm repositories, are similarly affected. Inaccurate understanding of the patterns of genetic structure in natural populations can result in incomplete collection strategies and a failure to archive the standing stock of co-adapted gene complexes from nature [6]. In ecology, precise inference of population structure is necessary to accurately describe the distribution of biodiversity across ecosystems, to improve conservation efforts, and, more generally, to further understanding of the evolutionary process.

Population structure also has important medical implications [7], [8]. A phenotypic trait, e.g. disease susceptibility, and an allelic state can become statistically associated over time due simply to their common occurrence in a reproductively cohesive subpopulation, rather than any causal mechanism. Thus, the false positive rate is often unacceptably high in associative genetics studies unless explicit corrections for population structure are built into the statistical models [e.g. 9], [10]. In medicine, accurate inference of population structure is necessary to control Type I error in association mapping studies that seek to identify the genes responsible for diseases that frequent particular human subpopulations.

The distinct needs of human disease research and ecological genetics have spurred the development of sensitive methods for identifying cryptic population structure [11]–[14]. Foremost among these new methods for population structure inference is the Bayesian Markov chain Monte Carlo (MCMC) method that has been deployed in the software STRUCTURE [11]. Pritchard et al. [11] recognized the need for a “natural” clustering procedure—an objective procedure that revealed groups directly from genetic polymorphism data, rather than relying on subjective, *a priori* notions of existing structure. Their method is thus distinguished from many of the classic statistical procedures for understanding genetic subdivision, such as F-statistics [15] and analysis of molecular variance [16]. Pritchard et al.'s [11] method relies on the assumption (predicted by the Wahlund effect) that, if population structure exists, then the mean deviation from Hardy-Weinberg and linkage equilibrium across a sampled population should be less given an assemblage of discrete subpopulations than if the population as a whole was treated as a single unit. Accordingly, the procedure maximizes Hardy-Weinberg and linkage equilibrium within K subpopulations by swapping individuals among them during the progression of a Markov chain. Sampling from the chain reveals the posterior probability of assigning each individual to each of the K subpopulations.

While assignment of individuals to subpopulations is automatic under Pritchard et al.'s [11] model, the determination of K is not. Strategies for estimating K have been proposed [17]–[19], but most require many time-consuming STRUCTURE runs [see 12], [13], [20 for alternative approaches]. A major innovation, in which the model propagated by the Markov chain treats K as a random variable, was offered by Pella and Masuda [21]. This model, which allows the number of subpopulations to be estimated alongside the assignment of individuals during the progression of a single Markov chain [22], has been implemented in the software STRUCTURAMA (available at http://www.structurama.org).

Another approach long used to reveal population structure without *a priori* specification of subpopulations is ordination, most typically in the form of principal component analysis (PCA) or principal coordinate analysis (PCO). These methods saw early uses in numerical taxonomy [23] and have since become a mainstay in population genetics studies of wild species. Ordination is most commonly used to decompose complex multilocus data sets into two or three dimensional scatter plots that represent genetic structure spatially, with putative subpopulations forming distinct clusters of points. However, the development of statistically rigorous methods to assign individuals to subpopulations using ordination results has been problematic. The role of ordination has largely been limited to informal visual corroboration of pre-existing ideas about population structure.

There has been a resurgence of interest in ordination in human genetics, where it has been promoted as a computationally-efficient, sensitive, and model-free alternative to Bayesian MCMC methods. Bauchet et al. [24] demonstrated the sensitivity of PCO for revealing subtle structure among European linguistic groups, results corroborated using STRUCTURE. Patterson et al. [25] designed statistical tests, using PCA, for the existence of population structure in a data set and for the number of significant principal component axes. Price et al. [14] used statistically significant axes to continuously adjust genotypic and phenotypic scores along an “ancestry eigenvector” prior to association analysis. Liu and Zhao [26] coupled ordination and cluster analysis to automatically assign individuals to K subpopulations. The latter idea is not new [27], [28], nor was extensive empirical validation provided, but the technique has been successfully used to identify a minimal subset of loci useful for assigning individuals of unknown origin to established human subpopulations [29].

In this study, we describe the development of a method, termed PCO-MC, which uses PCO followed by a statistically rigorous density clustering procedure (“modal clustering”) to infer population structure. PCO is a natural choice because of a long history of reliable results, its ability to accept missing data, and its computational efficiency. The development of the method relies on an examination of the properties of real data under PCO. We then discuss the capabilities of the procedure and provide guidelines for interpreting results using exemplar data sets. Finally, we compare the performance of PCO-MC with another automatic assignment procedure, the Bayesian MCMC approach implemented in STRUCTURAMA, using an extensive series of simulated data sets.

## Materials and Methods

### Properties of real data in principal coordinate analyses

Twenty four data sets containing multilocus amplified fragment length polymorphism (AFLP) genotypes, and three data sets containing inter-simple sequence repeat (ISSR) genotypes, were obtained (Table 1). Genotypic data had been scored as binary presence/absence characters by the original authors, and the data matrices varied in size from 12 to 506 samples and 30 to 2810 loci. Data sets sampled genetic variation across various taxonomic levels, from within-population to between-species. These dominant data sets were used to facilitate development of the PCO-MC method (via exploration of the properties of real genotypic data subjected to principal coordinate analysis), and to demonstrate some useful properties of PCO-MC.

**Table 1:** Real data sets used.

Organism	Marker system	Samples	Loci	Citation
Eubacteria:Proteobacteria:*Salmonella*	AFLP	19	166	[55]
Eubacteria:Proteobacteria:*Salmonella*	AFLP	72	176	[55]
Eukaryota:Viridiplantae:eudicotyledons:*Cardamine*	AFLP	86	359	[56]
Eukaryota:Viridiplantae:eudicotyledons:*Cicer*	ISSR	43	150	[57]
Eukaryota:Viridiplantae:eudicotyledons:*Coffea*	ISSR	15	230	[58]
Eukaryota:Viridiplantae:eudicotyledons:*Helianthus*	AFLP	62	91	[59]
Eukaryota:Viridiplantae:eudicotyledons:*Humulus*	AFLP	159	555	Reeves and Richards, unpublished
Eukaryota:Viridiplantae:eudicotyledons:*Lathyrus*	AFLP	37	210	[60]
Eukaryota:Viridiplantae:eudicotyledons:*Trollius*	AFLP	34	185	[61]
Eukaryota:Viridiplantae:eudicotyledons:*Trollius*	AFLP	180	117	[62]
Eukaryota:Viridiplantae:eudicotyledons:*Mimulus*	AFLP	50	474	[47]
Eukaryota:Viridiplantae:eudicotyledons:*Pritzelago*	AFLP	76	674	[52]
Eukaryota:Viridiplantae:eudicotyledons:*Veronica*	AFLP	207	583	[46]
Eukaryota:Viridiplantae:Filicopsida:*Polystichum*	AFLP	28	230	[63]
Eukaryota:Viridiplantae:Liliopsida:*Calopogon*	AFLP	60	468	[64]
Eukaryota:Viridiplantae:Liliopsida:*Carex*	AFLP	67	1394	[65]
Eukaryota:Viridiplantae:Liliopsida:*Conostylis*	AFLP	36	192	[66]
Eukaryota:Viridiplantae:Liliopsida:*Dupontia*	AFLP	121	162	[67]
Eukaryota:Viridiplantae:Liliopsida:*Elymus*	AFLP	161	1265	[68]
Eukaryota:Metazoa:Arthropoda:Decapoda:*Penaeus*	AFLP	26	443	[69]
Eukaryota:Metazoa:Chordata:Teleostei:*Brienomyrus*	AFLP	62	2810	[70]
Eukaryota:Metazoa:Chordata:Aves:*Larus*	AFLP	109	209	[71]
Eukaryota:Metazoa:Fungi:Ascomycota:*Gibberella*	AFLP	506	30	[72]
Eukaryota:Metazoa:Fungi:Ascomycota:*Macrophomina*	AFLP	24	312	[73]
Eukaryota:Metazoa:Fungi:Ascomycota:*Phialocephala*	ISSR	32	57	[74]
Eukaryota:Metazoa:Fungi:Basidiomycota:*Ustilago*	AFLP	12	207	[75]
Eukaryota:Metazoa:Mollusca:Bivalvia:*Anodonta*	AFLP	104	67	[76]

NTSYS 2.11x (Exeter Software) was used to calculate principal coordinates. Pairwise genetic distances were calculated using Jaccard's coefficient, appropriate for binary, multilocus data [30]. The resulting matrix was double-centered using the DCENTER module, then EIGEN was used to compute principal coordinates along all axes. Coordinate values were weighted by multiplying them by the percent variation explained by the axis to which they belonged.

Cluster analysis was performed using the MODECLUS procedure in SAS 9.1 (SAS Institute, Cary, NC). In PROC MODECLUS, a valley-seeking procedure identifies clusters as those groups of individuals that occur in regions of high principal coordinate density surrounded by regions of low density. All principal coordinate axes can be considered simultaneously. PROC MODECLUS uses kernel density estimation to generate an idealized, multidimensional density landscape (technically, the smoothed hyperdimensional probability density function) from which coordinate values were assumed to have been sampled. Density is estimated using a smoothing parameter (R) that corresponds to the radius of the hyperspherical uniform kernel. One can imagine moving a hollow sphere (the kernel) throughout principal coordinate space, stopping frequently to count the number of points within it, then calculating density as the number of points divided by the kernel volume. That density value is then assigned to the point in principal coordinate space upon which the kernel is centered. This produces a numerically smooth function from which peaks in density (clusters) can be defined by finding the valleys of low density between them.

During an analysis, R was varied from a small value that returned many clusters to a large value that returned one cluster. In total, 100 fixed-radius R values, spaced in even increments, were used to sample what we call “R-space,” or the union of all possible probability density functions for a data set. All analyses comprehensively sampled “informative R-space” (the subset of density landscapes that yield more than one and less than N [the number of individuals] clusters).

The weighted principal coordinate data from all 27 real data sets were processed using PROC MODECLUS with the following options: METHOD = 6, CASCADE = 1. PROC MODECLUS can perform a saddle test to determine whether a given cluster is significantly distinct. Details of the saddle test are available in the SAS manual (Chapter 42, The MODECLUS Procedure, SAS Institute, Cary, NC). We post-processed the SAS output to determine the number of times that each unique cluster was recovered across the replicated analyses, as well as the associated p-values. A value, termed “stability”, was calculated for each unique cluster as the percent of informative R-space where the cluster was found. Hence, two metrics related to the veracity of a particular cluster, p-value (from the saddle test) and stability, were obtained and their utility could be compared. A computer program that produces the SAS commands necessary to perform an analysis, and post-processes the SAS output for easy interpretation, is available (http://lamar.colostate.edu/~reevesp/PCOMC/PCOMC.html).

### Evaluation of performance using simulated data

To compare the performance of PCO-MC with the Bayesian approach of Pritchard et al. [11] as modified by Huelsenbeck and Andolfatto [22], we used simulated data. Following the strategy of Huelsenbeck and Andolfatto [22], data sets were simulated using the software *ms* [31], which uses an infinite-sites model under the coalescent to generate highly polymorphic, co-dominant data. 100 data sets were simulated under a symmetric island model with 76 different sets of parameters. An island model is appropriate because we are most concerned with circumscription, i.e. a discrete assignment of individuals into groups. Since discrete clusters are not necessarily the predicted product of other, more complex, demographic processes (e.g. isolation by distance or progenitor-derivative relationships), we have not modeled those important scenarios here, and leave them for future studies. The model parameters varied were: number of subpopulations (1, 2, 4, or 10); mutation rate (θ = 4N_0_μ, where N_0_ = diploid subpopulation size and μ = neutral mutation rate); and migration rate (M = 4N_0_m, where m = the fraction of each subpopulation made up of new migrant genotypes each generation). Four mutation rates (θ = 0.5, 1, 2, 4) and six migration rates (M = 0.5, 1, 2, 4, 8, 16) were used. Two very high migration rates (M = 8, 16), equivalent to two or four migrants per subpopulation per generation, were included to define the limits of resolution.

The data simulated with *ms* were converted to diploid data sets for STRUCTURAMA using a Perl script provided by Peter Andolfatto. Note that the empirical data sets described earlier contained dominant AFLP and ISSR genotypes while the simulated data sets were co-dominant. This discordance was necessary because, at present, there is no Bayesian MCMC procedure for automatic assignment that treats dominant data properly (STRUCTURE accepts dominant data, but K must be determined manually), and methods for population structure inference are typically compared using co-dominant data. All data sets contained 100 individuals. A total of 100 data sets were assembled per model, and all 76 models were addressed twice: once with 10 co-dominant loci per genotype, and again with 100. In total, 15,200 data sets that spanned the spectrum from highly structured (with high θ and low migration) to highly admixed (with low θ and high migration) to panmictic (with one unstructured population) were produced.

STRUCTURAMA analyses of the simulated data proceeded as described previously [22]. A single MCMC chain was run for 100,000 cycles, 12,500 of which were used as the burn-in period. The number of subpopulations was treated as a random variable following a Dirichlet process prior (“numpops = rv”) and the prior mean of the number of subpopulations was fixed at two (“expectedpriornumpops = 2”). Accuracy of inference is relatively insensitive to misspecification of this prior, thus a fixed value is acceptable [22]. 3500 post burn-in samples from the chain were used to calculate the mean partition, which was then compared with the expected partition as defined in the *ms* simulations.

PCO-MC analyses of the simulated data proceeded as described for the real data sets with a few exceptions. The shared band similarity index [32] (the “BAND” coefficient in NTSYS) was used to calculate genetic distances for the co-dominant data. The assignment was determined using a stability cutoff of 15% and a p-value cutoff of 0.9999 (p-values were essentially ignored). Classic measures of population subdivision were also computed for all data sets. F_st_ was calculated as θ_p_ [33] using GDA 1.1 [34]. G'_st_, a standardized measure of genetic differentiation that represents the proportion of the maximum differentiation possible for a given level of subpopulation homozygosity, was calculated using Hedrick's [35] equation 4b with values from FSTAT 2.9.3 [36].

Huelsenbeck and Andolfatto [22] measured accuracy as the average distance of the sampled partitions to the true partition using a metric described by Gusfield [37], the partition distance. Because PCO-MC does not produce a partition, but rather a more generic assignment that may contain nested sets of clusters and unassigned individuals, the partition distance could not be used. A correct inference was declared to have occurred whenever the membership within an inferred subpopulation was precisely that specified by the *ms* model used to simulate the data. Accuracy was measured as the number of subpopulations correctly identified across all 100 replicate data sets divided by the total number of correct inferences possible under the given model (e.g. when the number of subpopulations was four, the number of correct inferences possible across the 100 replicate simulated data sets was 400). The resulting value is the mean probability of success.

A Type I error occurred whenever a cluster was found that did not contain precisely the membership specified in the simulation model. The Type I error rate was calculated by dividing the total number of Type I errors incurred across the 100 replicate data sets by the number of correct inferences, a value proportional to the probability that any single inferred cluster was not a true subpopulation. Type II error occurred whenever a subpopulation known to exist was not identified. In this study the calculation of the probability of Type II error was straightforward: equal to one minus the mean probability of success. Because the relationship is deterministic, Type II error is not discussed further.

## Results and Discussion

### Properties of real data in principal coordinate analyses

PCA and PCO results have commonly been interpreted by simple visual inspection of plots of points along the first two or three axes. Although inferences obtained in this manner often agree with preconceived notions of genetic structure, there are two fundamental problems. First, the practice is subjective. Patterns can be deceiving, especially when plots are enhanced with additional visual information such as outlines, or when the point marker is varied according to *a priori* ideas of population structure. Thus, an objective procedure that is methodologically consistent with the traditional, strictly visual means of interpreting PCO output is needed. Second, the first two or three axes may explain only a small proportion of the total variation in a data set. On average, only 33% (sd = 16.1%) of the variation was explained by the first three axes for the 27 real data sets we examined. A rule of thumb in ordination is to consider axes until 70% to 90% of the total variation has been explained [38]. More than 20 principal coordinate axes would be needed to meet the 70% threshold for half of the real data sets we analyzed. Therefore, any method for interpreting ordination analyses of multilocus data should be capable of considering many or all axes simultaneously. The visual inspection method is inadequate in this respect. An algorithmic approach that clusters individuals using peaks and valleys in a multidimensional density landscape satisfies both concerns.

**Figure 1:**
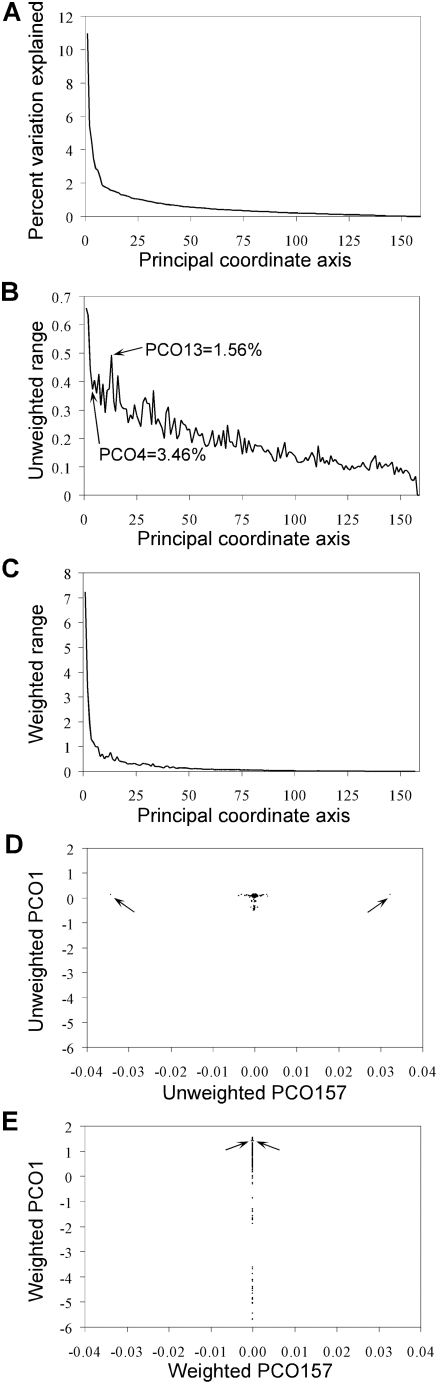
Weighting scheme to improve density estimation. A) Percent of total variation explained by each principal coordinate axis for an exemplar data set from *Humulus lupulus*. B) Before weighting, high order axes may contribute disproportionally to the dispersion of points along an axis. One problematic pair of axes (PCO4 and PCO13) is indicated. C) After weighting, the maximum distance between points better reflects the importance of the axis. D) Before weighting, two outliers present along PCO157 could detrimentally affect the analysis because density estimation only considers dispersion between points, not axis importance. E) After weighting, the spatial information present in inconsequential axes exerts little influence. Arrows mark the position of the two outliers.

An important technical issue is whether principal coordinate values can be used directly, without transformation, for density estimation. Principal coordinate values typically do not follow a normal distribution. This is not a problem because neither the density-based clustering algorithm, nor the non-parametric tests of significance implemented in PROC MODECLUS have distributional assumptions. But, it would be preferable if the dispersion of points along an axis accurately reflected the importance of that axis for explaining variation in the data set. This will generally be true without transformation because, by definition, low order axes hold the greatest variance (Figure 1A)—high variance means greater dispersion of points, and thus greater resolving power, along low order axes. But range is an important component of dispersion as well and the range of values encountered decreases erratically from low to high order axes (Figure 1B). This could cause the undesirable scenario where a high order axis of little significance exerts a detrimental effect on density estimation due to a few outlying values (Figure 1D). Rather than making an explicit determination of which axes hold signal and which hold noise (*contra* [25]), we have used a simple weighting scheme whereby a coordinate value is multiplied by the percent of variation explained by the axis to which it belongs. The weighted range presents a much more uniform decline (Figure 1C), akin to that observed for the variance. Likewise, the potential negative effect of outlying values is mitigated by this transformation (Figure 1E).

**Figure 2:**
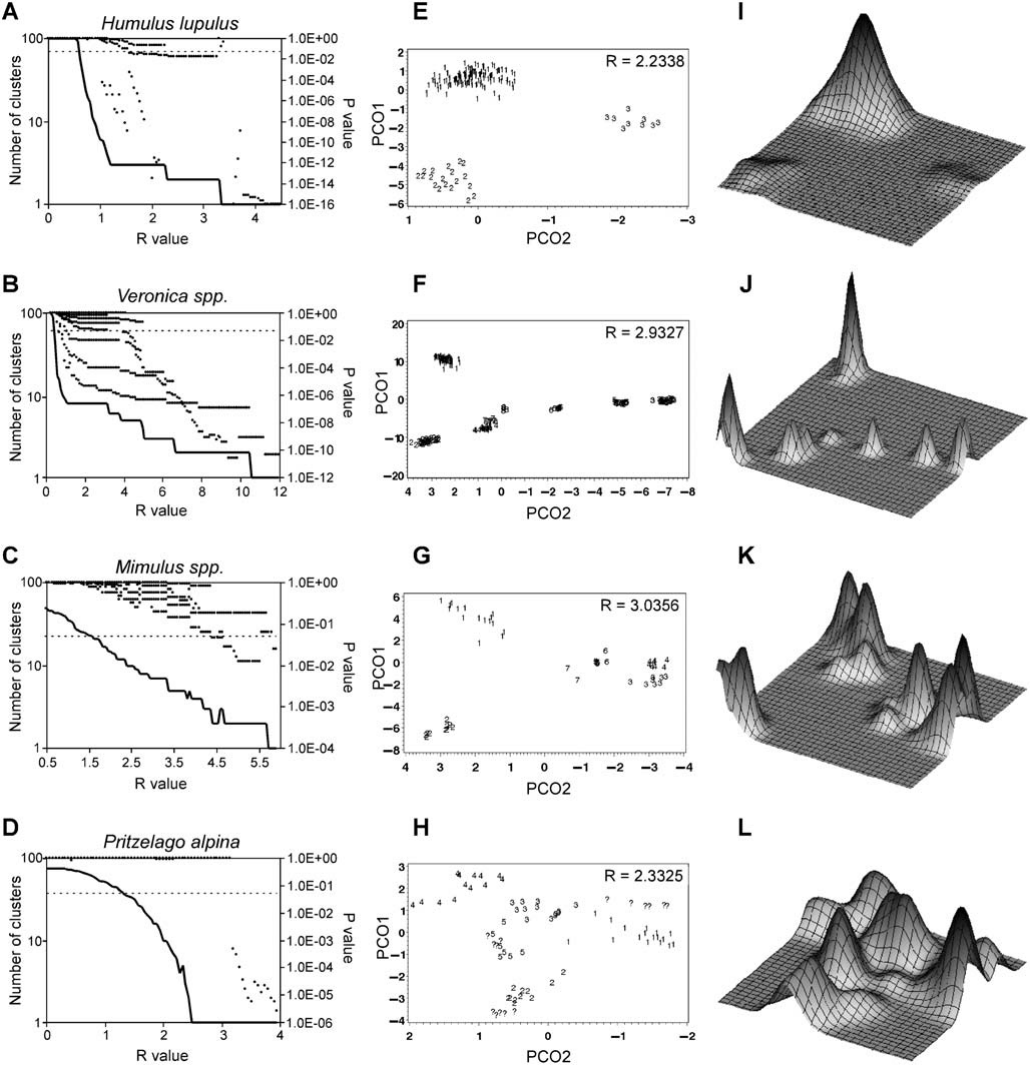
Density clustering of principal coordinates using four exemplar data sets. A–D) Relationship between p-value and the number of clusters inferred across R-space. Significant p-values (points below dotted line at p = 0.05) are found when cluster number (solid line) is insensitive to changes in R. E–H) Plots of first two principal coordinate axes. The positions of individuals are labeled according to the assignment made by PCO-MC for the single value of R shown, but using all possible axes. I–L) Representative density landscapes inferred from the first two principal coordinate axes and a single R value.

Thus, the PCO-MC approach uses weighted coordinate values from all axes simultaneously to estimate a multidimensional density landscape (see Figures 2I–L for representative landscapes). The precise form of the density landscape depends upon a smoothing parameter, R (see Table S1 for animations). Ultimately, R determines the number of clusters found, cluster membership, and significance values. Rather than rely on methods for finding a single, globally optimal R [39], we sample numerous R-values, thereby preserving locally optimal solutions. For each R, cluster membership and the associated p-value are noted. The support for the existence of a particular cluster can then be presented as a p-value, or as a stability value calculated as the frequency of occurrence of that cluster across a range of R-values.

The stability-based support metric appears more sensitive and better able to reveal subtle population structure than p-values. To accumulate stability, a cluster merely needs to be inferred repeatedly across a range of R-values. To achieve statistical significance, a cluster must first be inferred, then receive a significant p-value, a condition dependent upon the particular value of R used, variation within the cluster, and the total number of clusters. Moreover, while both support metrics were biased (larger clusters received better support values), the statistical association between p-value and cluster size was much stronger (R^2^ = 0.4258, p<0.0001 vs. R^2^ = 0.1417, p = 0.0153). Regression analysis suggested that 20+ individuals should be sampled per putative subpopulation to expect a p-value less than 0.05 (Figure S1). On the contrary, minimum sample sizes are not required to achieve high stability. Therefore, to avoid sampling restrictions, and to better discriminate between real and artifactual subpopulations we prefer the stability-based support metric.

### Capabilities of PCO-MC and interpretation of results

In what follows we describe the application of PCO-MC to four exemplar data sets with varying levels of genetic subdivision, from highly structured to virtually unstructured. These data sets are used to demonstrate some desirable properties of the method.

#### Data set 1, *Humulus lupulus*

We produced a data set of AFLP genotypes from 159 native North American *Humulus lupulus* (hops) individuals. Three named taxonomic varieties were included (*lupuloides*, *neomexicanus* and *pubescens*), with sampling focused on 29 populations of var. *lupuloides* from the Great Plains. Strong genetic structure in this data set is evident, visualized as two plateaus in cluster number as R was varied (i.e. either two or three clusters were most commonly found across R-space, Figure 2A). A total of four clusters stable over more than one third of informative R-space were recovered. Three corresponded to the named taxonomic varieties; the fourth included both var. *lupuloides* and var. *neomexicanus*. All clusters were statistically significant except for var. *neomexicanus*, perhaps due to small sample size (n = 9). Thus, PCO-MC suggests that the important genetic discontinuities in native North American *Humulus* are between named varieties, and that there is little evidence for strong genetic structure within var. *lupuloides*.

**Figure 3:**
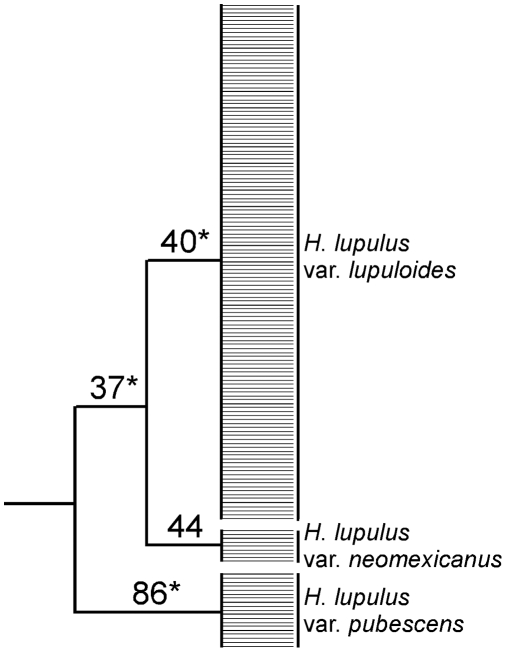
Hierarchical assignment resulting from PCO-MC analysis of *Humulus* data set. Numbers at nodes are stability values. Asterisks indicate statistically significant clusters (p<0.05).

PCO-MC may find sets of clusters that can logically be nested. This is distinct from the approach of Huelsenbeck and Andolfatto [22], which produces a mean partition with individuals uniquely assigned to one of K clusters, or the approach of Pritchard et al. [11] with admixture, where the probability of assignment of each individual to each cluster is estimated (although hierarchy can be imposed by iterating across multiple values of K [40]). For simplicity of display, we arrange the nested sets from PCO-MC into a tree [41], [42]. The PCO-MC assignment for the *Humulus* data, which produced four clusters that could be nested, is shown in tree form in Figure 3. Until studies can be undertaken to determine whether such hierarchical arrangements accurately represent hierarchical population structure, we recommend that they be regarded simply as a tool to provide a quick visual summary of support for the clusters found.

#### Data set 2, *Veronica spp*

PCO-MC produces results consistent with current understanding. A series of detailed studies showed sharp genetic discontinuities between populations of western Mediterranean *Veronica* subgenus *Pentasepalae*, in spite of limited morphological divergence and widespread homoplasy [43]–[46]. We re-analyzed the AFLP data set of Martínez-Ortega et al. [46], which includes 207 individuals sampled from 62 wild populations in Spain and Morocco, using PCO-MC. Our results confirmed their conclusions. PCO-MC identified as clusters the same eight taxa first described by Martínez-Ortega [43] using morphological and cytogenetic data (Figure S2A). While the eight clusters are easily observed in a plot of the first three principal coordinates (2D, Figure 2F; 3D not shown), it is important to point out that their inference was automatic within the PCO-MC procedure. In less obvious cases, like those that follow, speculation about cluster number and membership can be avoided.

#### Data set 3, *Mimulus spp*

PCO-MC can be used to reveal novel hypotheses or test existing hypotheses. Beardsley et al. [47] studied relationships among seven species of *Mimulus* section *Erythranthe* from the western United States. Within section *Erythranthe*, biological barriers to reproduction among species are often weak; however, strong ecological and geographical barriers to gene flow are in place [48]–[50]. In contrast to *Veronica*, where morphologically indistinguishable populations showed genetic structuring consistent with complete reproductive isolation, these *Mimulus* species exhibit substantial morphological divergence despite the possibility of low-level gene flow among them.

PCO-MC identified clusters containing *M. cardinalis* (cluster 1, Figure 2G), *M. parishii* (cluster 5), the Northern race of *M. lewisii* (cluster 3), and the Sierra Nevada race of *M. lewisii* (cluster 2). Two individuals described as “intermediates” between Sierran and Northern *M. lewisii* (cluster 7) appeared in a central position between their parents when the first three axes and a single R value were used, and were appropriately left unassigned when all coordinate axes were considered (Figure S2B). These results are consistent with Beardsley et al. [47]. In contrast, we found no evidence that *M. verbenaceus* and *M. eastwoodiae* are distinct from one another (cluster 4), a discrepancy possibly due to small sample size. But, we note that *M. verbenaceus* and *M. eastwoodiae* have contiguous, if not overlapping, ranges on the Colorado Plateau, are both hummingbird pollinated, and are fully crossable with no apparent reduction in F1 fitness [48]. Further sampling could resolve whether *M. verbenaceus* and *M. eastwoodiae* form a single genetically homogeneous group with two morphologically divergent phenotypes, or whether they are discrete evolutionary lineages.

Beardsley et al. [47] found that the Sierran and Northern races of *M. lewisii* were sister taxa. This, plus the observation of intermediate individuals in the wild, led them to retain *M. lewisii* as a single species. Vickery and Wullstein [48], noting substantial divergence in floral and vegetative morphology, isozymes, and petal pigmentation chemistry, as well as moderate postzygotic barriers to gene flow, suggest that the two races could be construed as two species. PCO-MC found no association between the Sierran and Northern races of *M. lewisii*. The clusters are as distant from one another in principal coordinate space as any two species considered (clusters 2 and 3). The sister-taxon relationship found by Beardsley et al. [47] may be an artifact of the application of bifurcating trees to populations with a history of limited gene exchange [51]. Thus, the results of PCO-MC were consistent with Vickery and Wullstein [48]: the genetic discontinuity between races of *M. lewisii* is sufficient for their consideration as separate species.

#### Data set 4, *Pritzelago alpina*

PCO-MC does not find structure when none exists. While this property is best demonstrated using simulated data (below), we present a real example here. Kropf et al. [52] studied the high alpine plant *Pritzelago alpina* in the disjunct mountain ranges of central and southern Europe. Their thesis was that European alpine species should show less population subdivision than lowland or montane species with the same continental distribution. This “displacement refugia model” was based upon predicted changes in available habitat during repeated cycles of glaciation. Using AMOVA and a neighbor-joining tree-based analysis, Kropf et al. [52] claimed evidence for significant structuring of genetic diversity in *P. alpina* into four or five geographical regions. However, citing poor support for basal relationships among populations from different geographical regions in the neighbor-joining tree, and the lack of resolution of distinct groups in a principal coordinate analysis, they concluded that the AFLP data were consistent with their thesis.

Reanalysis of the Kropf et al. [52] AFLP data set using PCO-MC provided no support for any geographic structuring of genetic diversity within *P. alpina*. There were no plateaus in cluster number across R-space (Figure 2D), no statistically significant clusters, and only a few stable clusters (which corresponded to single populations or geographically disjunct assemblages of populations) (Figure S2C). Thus, PCO-MC supports the displacement refugia model but is inconsistent with some of their interpretations. One of the reasons for this inconsistency could be that their phylogenetic trees, which were built from population level data, may be over-resolved [51].

### Evaluation of performance using simulated data

**Figure 4:**
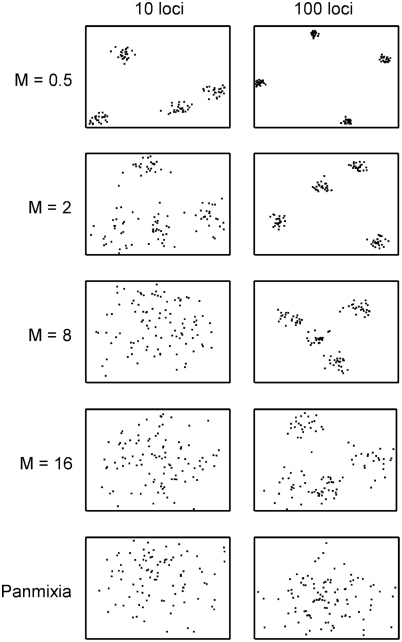
Range of variation and admixture encountered in simulated data sets as viewed by PCO. Number of subpopulations = 4 for all rows of graphs except the last, where a single, panmictic population was simulated. Migration rate (M) is specified at left. Mutation rate (θ) = 0.5. Representative plots of the first two principal coordinate axes are shown. When population structure is present, adding loci increases the density of points within a cluster, permitting more accurate inference.

**Figure 5:**
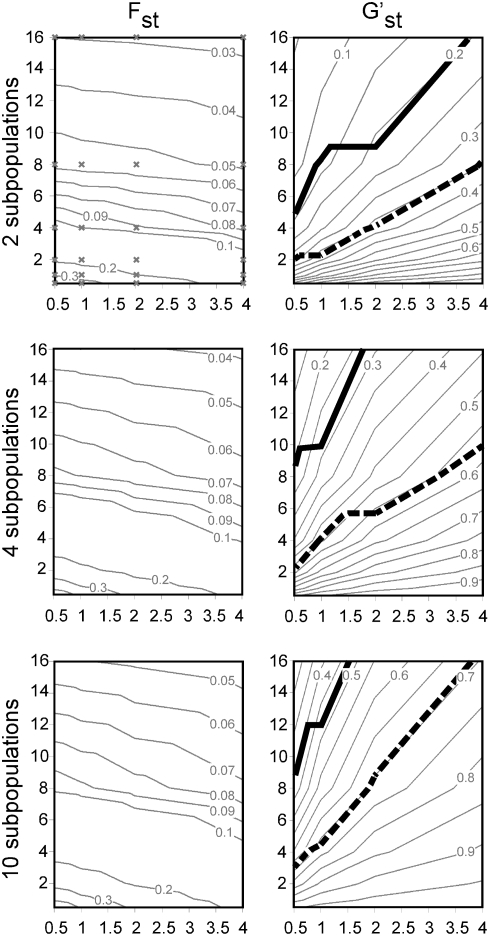
Levels of population subdivision in simulated data sets as viewed by F_st_ and G'_st_. X axes = mutation rate (θ), Y axes = migration rate (M). Contour plots were generated using interpolation between data sets sampled at the X's plotted in the top left panel. Bold solid lines in G'_st_ plots represent the 95% success contour for PCO-MC analysis using 100 loci, dashed lines are for K = rv. Below these lines the level of population subdivision was sufficient for correct identification of more than 95% of known clusters.

Because the true population structure is not generally known for real data sets sampled from nature, it is necessary to use simulated data to evaluate the performance of any genetic clustering procedure. Huelsenbeck and Andolfatto [22] have defined a series of data sets, extended here, that are useful for this purpose. By varying the mutation and migration rates in a coalescent model, a complex assemblage of data sets representing a broad range of population subdivision can be produced. The range of subdivision, considered from the perspective of PCO, and from F_st_ and G'_st_, is shown in Figures 4 and 5, respectively.

With the Bayesian MCMC approach of Huelsenbeck and Andolfatto [22], where K is treated as a random variable (hereafter, “K = rv”), Type I error control is inherent to the procedure. By analyzing data sets simulated with no population structure (i.e. derived from a single panmictic population) we estimated the probability of Type I error of their procedure to be 0.125%. With PCO-MC, Type I error control is accomplished by applying a post-analysis cutoff criterion to distinguish correct from incorrect inferences. We prefer to use a minimum stability value instead of a maximum p-value for this purpose. Across all simulated data sets, ninety-five percent of incorrect clusters had stability values less than 11%. Ninety-five percent of correct clusters had stability values greater than 16%. Thus, any stability cutoff value between 11% and 16% should provide adequate Type I error control (15% is optimal).

**Figure 6:**
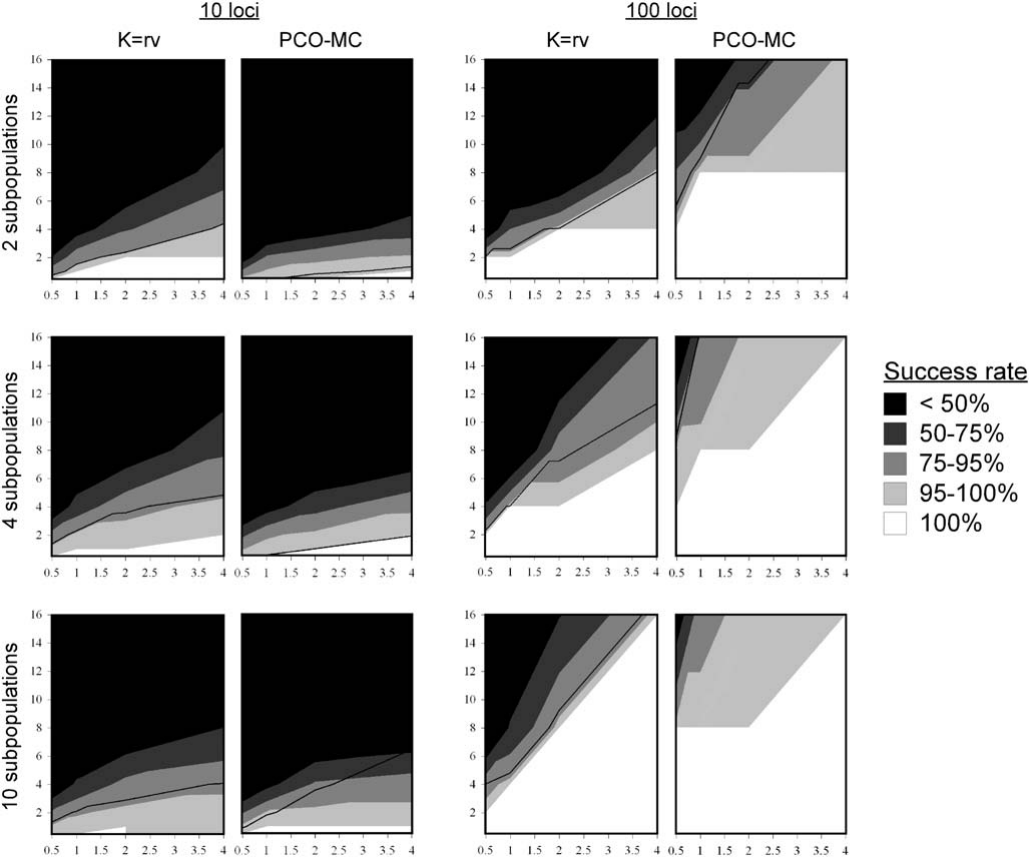
Relative performance of K = rv and PCO-MC methods for population structure inference using simulated data. X axes = mutation rate (θ), Y axes = migration rate (M). Contour intervals are shaded according to the probability of successful inference. Below the thin black line, Type I error was acceptable (α<0.05). Type I error was <0.05 for all combinations of θ and M when PCO-MC was applied to the 100 locus, 10 population data sets.

The success of PCO-MC and K = rv in retrieving the expected subpopulations is shown in Figure 6. For data sets with 10 loci, the K = rv method performed slightly better. As implied by Figure 4, PCO-MC's strength of inference is compromised when few loci are considered. When population substructure exists, adding loci focuses dispersed principal coordinate values into compact clusters of points.

Both methods, however, were relatively insensitive to subtle population subdivision when only 10 loci were sampled. We use the standardized population differentiation metric G'_st_ [35] to illustrate. Unlike F_st_, G'_st_ allows the unbiased comparison of population differentiation between data sets that differ in the level of within-subpopulation homozygosity. This is important here because variation in the mutation rate causes differences in the level of within-subpopulation homozygosity between *ms* simulation models. In contrast to F_st_, the G'_st_ contours are roughly isoclinal to the contours representing performance (Figure 5). Hence, for this study, G'_st_ is a consistent predictor of the magnitude of population subdivision necessary for a particular method to perform well, but F_st_ is not.

For K = rv and 10 loci, more than 95% of the correct clusters were returned when G'_st_>0.5 (F_st_>0.08), and for PCO-MC, when G'_st_>0.7 (F_st_>0.15). Values reported are minimum values, calculated using data sets with two subpopulations. Higher levels of subdivision were necessary for accurate inference as subpopulation number increased, possibly due to fewer individuals per subpopulation or to bias in the F_st_ and G'_st_ estimators. When the number of loci was increased to 100, the overall power of inference of both methods increased dramatically, but PCO-MC exhibited broadly superior performance over the simulation parameter space, both in terms of a higher probability of correct inference and a lower probability of Type I error (Figure 6). With 100 loci, PCO-MC proved to be substantially more sensitive than K = rv, providing >95% correct retrieval of subpopulations when G'_st_>0.2 (F_st_>0.03), whereas for K = rv, G'_st_ had to be greater than 0.35 (F_st_>0.05) to achieve similar performance (Figure 5).

There appears to be a relatively distinct lower limit on the level of population subdivision necessary before Bayesian MCMC methods, as a class, will yield accurate assignments. We find that the limit of resolution for the K = rv method of STRUCTURAMA (G'_st_>0.35; G_st_>0.025) is similar to that found for STRUCTURE [11] and BAPS [13] using a different simulation strategy [53]. Those methods began to break down in accuracy (<97% correct) with G'_st_<0.39 and G_st_<0.05. Accordingly, we predict that PCO-MC should outperform the methods of Pritchard et al. [11] and Corander et al. [13] as well, in cases where structure is subtle and many loci are available. We caution, however, that this prediction is based on simulated data, which may differ fundamentally from real data. In real data sets, the evidence of coancestry that manifests as faint genome-wide linkage disequilibrium may be augmented by factors that were not modeled here (e.g. natural selection and the organization of the genome into discrete chromosomes).

We find it surprising that a simple metric such as multilocus genetic distance, if properly transformed and interpreted, can produce extremely accurate inferences of subtle population structure. Importantly, when the probability of retrieval of correct subpopulations was high, the probability of retrieval of erroneous subpopulations was low. This relationship is not necessarily an expectation for a procedure like PCO-MC, which does not produce a partition (where Type I error and success rates are interdependent).

### Conclusion

The use of genetic data to assign individuals to subpopulations for ecological studies is a different goal from population structure-based correction of the false positive rate in associative genetics. If the purpose is to produce a description of intraspecific biodiversity, circumscription of natural groups is necessary, and a discrete assignment seems most useful. If the purpose is Type I error control for structured association mapping, a representation of population structure as a continuous or quasi-continuous variable (e.g. [14], [54]) is most appropriate. The results of this study suggest that, if the goal is circumscription, then Bayesian MCMC approaches should be reserved for small data sets, where they are accurate and computationally efficient. For large genome-scale data sets, we propose that highly-sensitive two-step procedures that couple ordination with clustering may be best.

## Supporting Information

Figure S1 Exponential regression of two support metrics (p-value and stability) on cluster size for 27 real data sets. Grey points indicate observed support values; triangles indicate median support values calculated when three or more clusters of a particular size were found. Regressions were performed using median values. A) P-value is strongly associated with cluster size. Dotted line indicates a p-value of 0.05. Twenty or more individuals per subpopulation should be sampled in order to achieve p<0.05. B) Stability value is weakly associated with cluster size. In general, smaller subpopulations can be inferred by using a stability based cutoff instead of a significance based cutoff.
(0.06 MB TIF)

Figure S2 Hierarchical assignments from PCO-MC analysis of three additional exemplar data sets. Numbers at nodes are stability values. Asterisks indicate clusters found to be statistically significant (p<0.05). A) *Veronica* spp. [46]. B) *Mimulus* spp. [47]. C) *Pritzelago alpina* [52].
(0.18 MB TIF)

Table S1 Animations demonstrating change in the density landscape, and thus the assignment, with changing R value.
(0.05 MB DOC)
