# Supplementary figures and images for: Accurate Inference of Subtle Population Structure (and Other Genetic Discontinuities) Using Principal Coordinates
Source: PLoS One. 2009 Jan 27;4(1):e4269. doi: 10.1371/journal.pone.0004269 (PMC2625398; doi:10.1371/journal.pone.0004269)

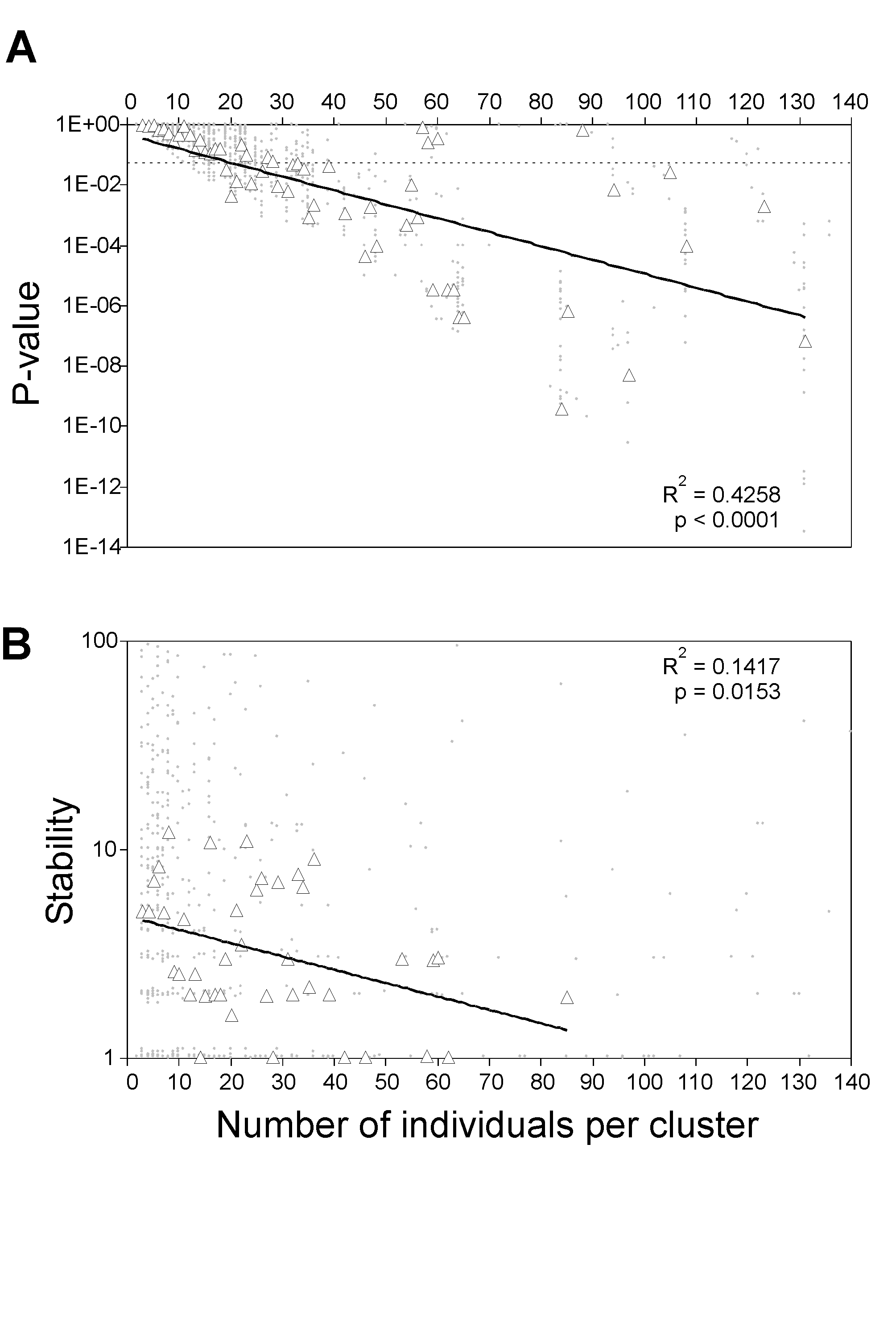

Supplement: Figure S1 — Exponential regression of two support metrics (p-value and stability) on cluster size for 27 real data sets. Grey points indicate observed support values; triangles indicate median support values calculated when three or more clusters of a particular size were found. Regressions were performed using median values. A) P-value is strongly associated with cluster size. Dotted line indicates a p-value of 0.05. Twenty or more individuals per subpopulation should be sampled in order to achieve p<0.05. B) Stability value is weakly associated with cluster size. In general, smaller subpopulations can be inferred by using a stability based cutoff instead of a significance based cutoff. (0.06 MB TIF) [file pone.0004269.s001.tif]
