# Supplementary material for: Accurate Inference of Subtle Population Structure (and Other Genetic Discontinuities) Using Principal Coordinates
Source: PLoS One. 2009 Jan 27;4(1):e4269. doi: 10.1371/journal.pone.0004269 (PMC2625398; doi:10.1371/journal.pone.0004269)
Supplement: Table S1 — Animations demonstrating change in the density landscape, and thus the assignment, with changing R value. (0.05 MB DOC) [file pone.0004269.s003.doc]

| Data set | Animation type | URL |
| --- | --- | --- |
| *Humulus lupulus* | Density landscape, 3D | http://lamar.colostate.edu/~reevesp/PCOMC/HopsSurface.gif |
|  | Density landscape, contour | http://lamar.colostate.edu/~reevesp/PCOMC/HopsContour.gif |
|  | Assignment | http://lamar.colostate.edu/~reevesp/PCOMC/Humulus.gif |
| *Veronica* spp. | Density landscape, 3D | http://lamar.colostate.edu/~reevesp/PCOMC/VeronicaSurface.gif |
|  | Density landscape, contour | http://lamar.colostate.edu/~reevesp/PCOMC/VeronicaContour.gif |
|  | Assignment | http://lamar.colostate.edu/~reevesp/PCOMC/Veronica.gif |
| *Mimulus* spp. | Density landscape, 3D | http://lamar.colostate.edu/~reevesp/PCOMC/MimulusSurface.gif |
|  | Density landscape, contour | http://lamar.colostate.edu/~reevesp/PCOMC/MimulusContour.gif |
|  | Assignment | http://lamar.colostate.edu/~reevesp/PCOMC/Mimulus.gif |
| *Pritzelago* *alpina* | Density landscape, 3D | http://lamar.colostate.edu/~reevesp/PCOMC/PritzelagoSurface.gif |
|  | Density landscape, contour | http://lamar.colostate.edu/~reevesp/PCOMC/PritzelagoContour.gif |
|  | Assignment | http://lamar.colostate.edu/~reevesp/PCOMC/Pritzelago.gif |
